# Afzelin attenuates asthma phenotypes by downregulation of GATA3 in a murine model of asthma

**DOI:** 10.3892/mmr.2015.3391

**Published:** 2015-02-26

**Authors:** WENBO ZHOU, XIUHONG NIE

**Affiliations:** Department of Respiratory Diseases, Xuanwu Hospital, Capital Medical University, Beijing 100053, P.R. China

**Keywords:** asthma, immunoglobulin E, airway hyperresponsiveness, T-helper 2, eosinophil, GATA-binding protein 3

## Abstract

Asthma is a serious health problem causing significant mortality and morbidity globally. Persistent airway inflammation, airway hyperresponsiveness, increased immunoglobulin E (IgE) levels and mucus hypersecretion are key characteristics of the condition. Asthma is mediated via a dominant T-helper 2 (Th2) immune response, causing enhanced expression of Th2 cytokines. These cytokines are responsible for the various pathological changes associated with allergic asthma. To investigate the anti-asthmatic potential of afzelin, as well as the underlying mechanisms involved, its anti-asthmatic potential were investigated in a murine model of asthma. In the present study, BALB/c mice were systemically sensitized using ovalbumin (OVA) followed by aerosol allergen challenges. The effect of afzelin on airway hyperresponsiveness, eosinophilic infiltration, Th2 cytokine and OVA-specific IgE production in a mouse model of asthma were investigated. It was found that afzelin-treated groups suppressed eosinophil infiltration, allergic airway inflammation, airway hyperresponsiveness, OVA-specific IgE and Th2 cytokine secretion. The results of the present study suggested that the therapeutic mechanism by which afzelin effectively treats asthma is based on reduction of Th2 cytokine via inhibition of GATA-binding protein 3 transcription factor, which is the master regulator of Th2 cytokine differentiation and production.

## Introduction

Asthma is a chronic inflammatory airway disease. It affects over 300 million individuals worldwide with an expected increase of 100 million by 2025 (1,2). The pathophysiological characteristics of allergic asthma, including chronic pulmonary eosinophilia, airway hyperresponsiveness (AHR) to a variety of nonspecific spasmogenic stimuli, excessive airway mucus production and elevated serum immunoglobulin E (IgE) levels are all associated with aberrant T-helper 2 (Th2) cell responses. Th2 cells are known to secrete interleukin (IL)-4, -5, -9 and -13. These cytokines, particularly IL-4, -5 and -13, have been documented to have a relatively important role in asthma progression. Th2 cell differentiation is driven by the transcription factor GATA-binding protein 3 (GATA-3), a member of the GATA family of zinc finger proteins (3). This transcription factor is known as the master regulator of Th2-cell differentiation. GATA-3 is suppressed by T-bet expressed in T cells, a Th1-specific transcription factor, which is hypothesized to induce interferon (IFN)-γ production while inhibiting IL-4 production (4).

**Figure 1:**
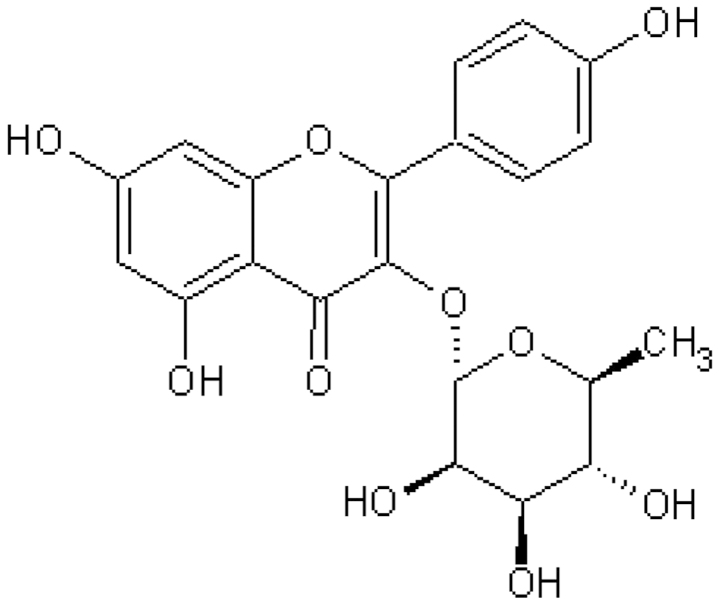
Structure of afzelin; 5,7-dihydroxy-2-(4-hydroxyphenyl)-3-[(2*S*, 3*R*,4*R*,5*R*,6*S*)-3,4,5-trihydroxy-6-methyloxan-2-yl] oxychromen-4-one); molecular mass, 432.38 g/mol.

Afzelin (Fig. 1) is a flavonol glycoside found in *Ficus palmata* and *Nymphaea odorata*. Previously, it has been found to inhibit lipid peroxidation and cyclooxygenase (COX)-1 and COX-2 *in vivo*. It is the rhamnoside of kaempferol, which has been documented to suppress inflammatory-cell infiltration in a mouse model of asthma (5). A previous study indicated that afzelin inhibits the growth of breast cancer cells through stimulating apoptosis, while being relatively non-toxic to normal cells (6). However, the effects of afzelin on asthma phenotypes have remained to be elucidated. The present study was performed to investigate the anti-asthmatic effect of afzelin and its mechanism of action in a mouse model of asthma.

## Materials and methods

### Experimental animals

A total of 30 female BALB/c mice (five weeks old, 25–30 g) were attained from the animal house of the Capital Medical University (Beijing, China), and maintained under controlled conditions, temperature (24±2°C), relative humidity (60±10%) and photoperiod (12-h light/dark cycle). The room was well ventilated (>10 air changes/h) with fresh air, as per the Committee for the Purpose of Control and Supervision on Experiments on Animals guidelines. Animals were fed on a standard pellet diet and sterilized water was provided *ad libitum*. Animals acclimated for seven days were used for the pre-clinical studies. Approval of the animal experimental protocols was obtained from the ethics committee of the Capital Medical University (Beijing, China).

### Reagents

Chicken egg albumin (OVA, grade V), aluminium hydroxide gel (alum) and dexamethsone (Dexa), acetyl-β-methylcholine chloride (methacholine) and protease inhibitor cocktail were purchased from Sigma-Aldrich (St. Louis, MO, USA). Antibodies used for western blotting were purchased from Cell Signaling Technology (Beverly, MA, USA). Afzelin (purity, 99%) was acquired from Chirochem (Daejeon, Korea). All other chemicals and reagents were commercially obtained from Sigma-Aldrich and were of the highest quality.

### Segregation of animals and dosing schedule

**Figure 2:**
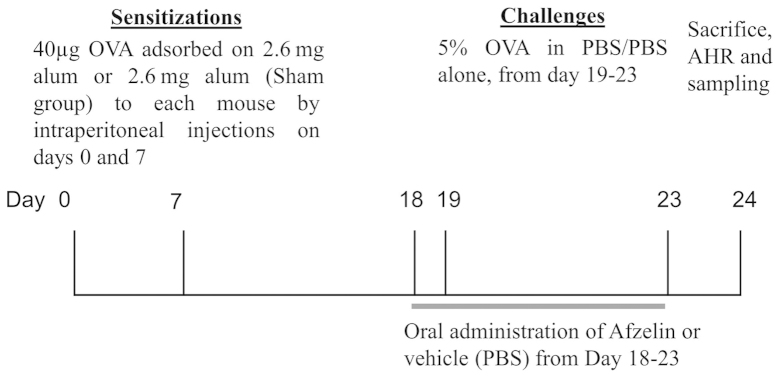
Experimental protocol for the induction of allergic asthma. Female BALB/c mice (5 weeks old) were grouped, sensitized and challenged. OVA, chicken egg albumin; PBS, phosphate-buffered saline.

Mice were segregated into six groups (six mice in each group) following acclimation; each group was termed according to sensitization/challenge/treatment: Group 1, SHAM/phosphate-buffered saline (PBS)/Vehicle (Veh; normal controls); group 2, OVA/OVA/Veh (OVA controls, OVA-sensitized and OVA-challenged); group 3, OVA/OVA/Dexa [OVA-sensitized, OVA-challenged and Dexa-treated (0.75 mg/kg)]; and groups 4–6, OVA/OVA/afzelin [OVA-sensitized, OVA-challenged and afzelin-treated (0.1, 1 and 10 mg/kg)]. The test compounds and the Dexa were administered orally, once daily from day 19 to day 23 (Fig. 2) (7). PBS was used as a vehicle.

### Sensitization, airway OVA challenging and treatment

The animals were sensitized intraperitoneally with 40 *μ*g OVA plus 2.6 mg aluminum hydroxide in 200 *μ*l PBS on days 0 and 7. Mice were then challenged from days 19 to 23 (5 min per day) with 5% OVA in PBS (OVA groups) or PBS (Sham/PBS/Veh) as described previously with certain modifications (8). Mice were administered the test drug and Dexa once a day from days 19 to 23. Mice were sacrificed on day 24 by heart puncture under ether anesthesia (Sigma-Aldrich), and bronchoalveolar lavage was performed to evaluate lung eosinophilia.

### Evaluation of AHR

AHR, in the form of airway resistance was estimated in anesthetized mice using the FlexiVent system (Synol High-Tech, Beijing, China), which uses a computer-controlled mouse ventilator and integrates with respiratory mechanics, as described previously (9). Final results were expressed as airway resistance with increasing concentrations of methacholine (Mch; 0, 2, 4, 8, 12 and 16 mg/ml).

### Bronchoalveolar lavage fluid (BALF) collection

After mice were bled and sacrificed following anesthesia with ether, BALF was collected for differential cell counting and measurement of cytokines. This was performed by cannulating the upper part of the trachea and lavaging three times with 0.5 ml PBS containing 0.05 mM EDTA (7). The BALF was centrifuged at 4,000 × g at 4°C for 3 min and the cells were separated from the fluid. The supernatant was stored at −70°C until use. The cells were re-suspended in PBS containing 0.05 mM EDTA and the total cell number was determined by using a hemocytometer. The differential BAL cells were counted using microscopy (MCL-3000; MCALON, Beijing, China) following cytospin preparations and Giemsa staining (Giemsa stain modified, Sigma-Aldrich).

### Cytokine measurement

Cytokine measurement was performed from serum samples of animals. Levels of cytokines IL-5, -13 and -4 as well as IFN-γ were determined using ELISA (R&D Systems, Minneapolis, MN, USA). The ELISAs were performed as per the manufacturer’s instructions.

### Measurement of OVA-specific IgE

Each well of a microtiter plate (Abcore, Ramona, CA, USA) was coated with 5 *μ*g OVA in 100 *μ*l PBS overnight at 4°C. Following three washes, nonspecific sites were blocked with 0.5% Tween 20 (Abcore) in PBS. Mouse sera in duplicate were added to the Ag-coated wells, the plates were incubated and bound IgE was detected with biotinylated anti-mouse IgE (BD Pharmingen, San Diego, CA, USA). Streptavidin-peroxidase conjugates (Takara Biotechnology Co., Ltd., Dalian, China) were added and the bound enzymes were detected with the addition of a tetramethylbenzidine substrate system (BD Pharmingen) and absorbance was read at 450 nm using an ultraviolet spectrophotometer (UV-3600; Shimadzu Corporation, Kyoto, Japan). Absorbance was converted to arbitrary units.

### Western blot analysis

The lungs were homogenized in a homogenizing buffer [1% NP-40, 150 mM NaCl, 50 mM 4-(2-hydroxyethyl)-1-piperazineethanesulfonic acid (Sigma-Aldrich), phenylmethylsulfonyl fluoride and complete protease inhibitor cocktail (Bio-Rad Laboratories, Inc., Hercules, CA, USA)]. Protein estimation of the samples was performed according to the Bradford method (10). For western blotting, 30 *μ*g protein was denatured at 100°C for 5 min in Tris-glycine SDS (Abcore) sample loading buffer. Protein samples were loaded onto 10% SDS gels and resolved at 70 V (300 mA) for 3 h and then electro-transferred onto a polyvinylidene difluoride membrane (Bio-Rad Laboratories, Inc.) in transfer buffer using a Mini Transblot electrophoretic transfer cell (Bio-Rad Laboratories, Inc.) for 90–120 min at 150 V. Membranes were blocked in 5% fat-free dry milk (Abcore) dissolved in Tris-buffered saline and Tween 20. for 2.5 h at room temperature. Anti-GATA3 and anti-T-bet mouse polyclonal antibodies (1:1,000 dilution; Bio-Rad Laboratories, Inc.) were used to determine expression of their corresponding proteins, and a monoclonal β-actin antibody was used as the loading control (Sigma-Aldrich) (11). After incubation with the primary antibodies overnight at 4°C the membranes were incubated with goat anti-mouse immunoglobulin G secondary antibody (1:5,000 dilution; Bio-Rad Laboratories, Inc.) for 1 h at 25°C. The blots were visualized with a chemiluminescent detection system (ECL; GE Healthcare Australia, Rydalmere, Australia) according to the manufacturer’s instructions.

### Histological examination

After BALF was obtained, the left lung was removed, fixed in 10% neutral buffered formalin for 24 h and then the specimens were dehydrated and embedded in paraffin in a standard manner. In order to perform histological examination, 5-*μ*m sections of fixed embedded tissues were cut and stained with hematoxylin & eosin (Abcore) according to routine laboratory procedures (12). Histological analyses were performed by pathologists blinded to the treatment groups. For each mouse, five airway sections, distributed throughout the left lung, were analyzed with the use of a light microscope (MCL-3000) attached to an image-analysis system (Image-Pro Plus 4.0; Media Cybernetics, Minneapolis, MN, USA). The images were then cropped and corrected for brightness and contrast, but otherwise were not manipulated (7).

### Statistical analysis

Groups were analyzed using a one-way analysis of variance followed by Dunnett’s multiple comparison tests to examine differences between OVA-challenged as well as PBS- and afzelin-and Dexa-treated groups. P<0.05 was considered to indicate a statistically significant difference. Values are presented as the mean ± standard error of the mean for each group.

## Results

### Afzelin decreases AHR in experimental asthma

**Figure 3:**
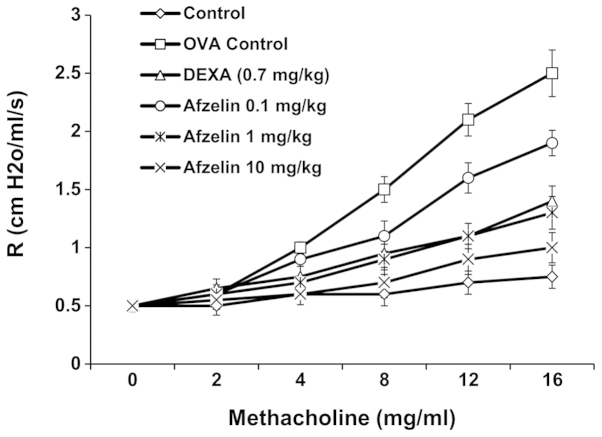
Measurement of airway hyperresponsiveness. Afzelin administration reduces airway hyperresponsiveness in mice as measured by a methacho-line dose-responsive cure for airway resistance. Values are expressed as the mean ± standard error of the mean. ^*^P<0.05, vs. sham and ^**^P<0.01, vs. sham (Student’s t test). Dexa, dexamethasone; OVA, chicken egg albumin.

To examine the effect of afzelin on AHR, airway resistance was measured in anaesthetized mice by invasive whole-body plethysmography. No significant difference was found in baseline airway resistance among the six groups. The airway resistance generated by administration of Mch at doses of 0–16 mg/ml significantly increased in the OVA and afzelin (0.1 mg/kg)-treated groups. However, the control, Dexa- and afzelin (1 and 10 mg/kg)-treated groups exhibited a sharp decrease in airway resistance (Fig. 3).

### Afzelin attenuates airway inflammation

**Figure 4:**
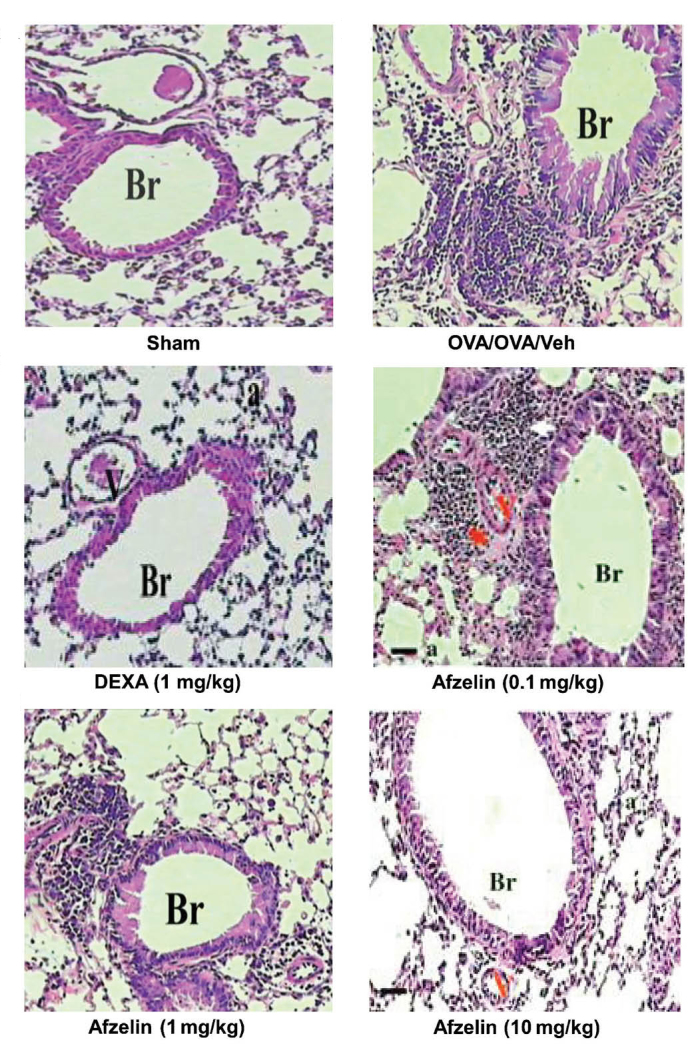
Afzelin treatment significantly reduced airway cellular infiltration as detected by hematoxylin & eosin staining of lung sections. Dexa, dexa-methasone; OVA, chicken egg albumin; Veh, vehicle; Br, bronchiole.

Apart from macrophages, only few inflammatory cells were detected in the control group. However, a significant increase in total cell number was observed in OVA-sensitized and challenged animals, when compared with those in the control mice. The effect of afzelin on allergen-induced inflammatory cell infiltration was assessed in animals treated with three different doses of afzelin. As shown in Table I, afzelin at 1 and 10 mg/kg suppressed allergen-induced inflammatory cell infiltration. However, in the case of the 0.1 mg/kg-treated group, infiltration of inflammatory cells was not reduced. The anti-inflammatory effect of afzelin was further demonstrated by histological examination of hematoxylin & eosin-stained sections (Fig. 4). A marked affluence of inflammatory cells into the airway was observed in OVA-sensitized/challenged mice, but not in the PBS-treated control mice. Mice treated with afzelin exhibited a marked diminution in inflammatory cell infiltration into the airways.

### Afzelin affects Th1 and Th2 cytokine release

**Figure 5:**
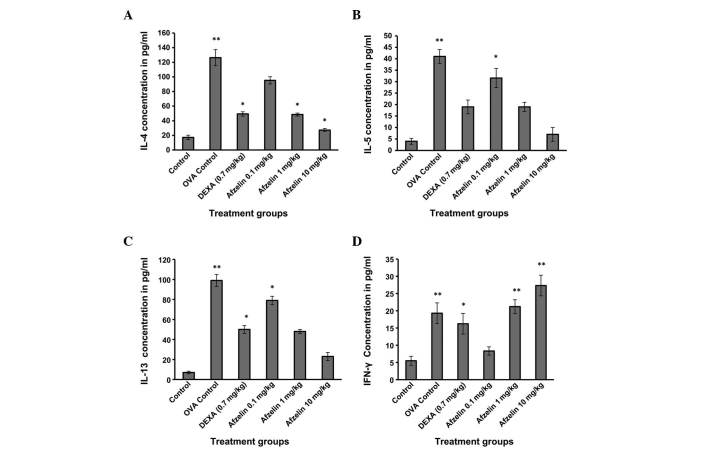
Effect of test compounds on Th2 cytokine release. (A) IL-4; (B) IL-5; (C) IL-13 and (D) IFN-γ. The levels of IL-4, IL-5 and IL-13 in BAL fluid were quantified by sandwich ELISA and expressed as picogram per milliliter. Values are expressed as the mean ± standard error of the mean. ^*^P<0.05, vs. sham and ^**^P<0.01, vs. sham (Student’s t test). TH2, T-helper 2; IL, interleukin; IFN, interferon; BAL, bronchoalveolar lavage; Dexa, dexamethasone; OVA, chicken egg albumin.

Measurement of the Th2 cytokines IL-4, -5 and -13 was performed in the serum collected from the mice. Mice treated with the test compound afzelin demonstrated no significant change in cytokine release when compared with those in the control at doses of 1 and 10 mg/kg (Fig. 5). However, cytokine levels measured in the 0.1 mg/kg-treated group of animals exhibited a significant variation from the control group. Afzelin increased the release of IFN-γ, a Th1 cytokine, indicating that it affects T-cell differentiation, which was further supported by its effect on GATA3 and T-bet.

### Afzelin reduces OVA-specific IgE levels

**Figure 6:**
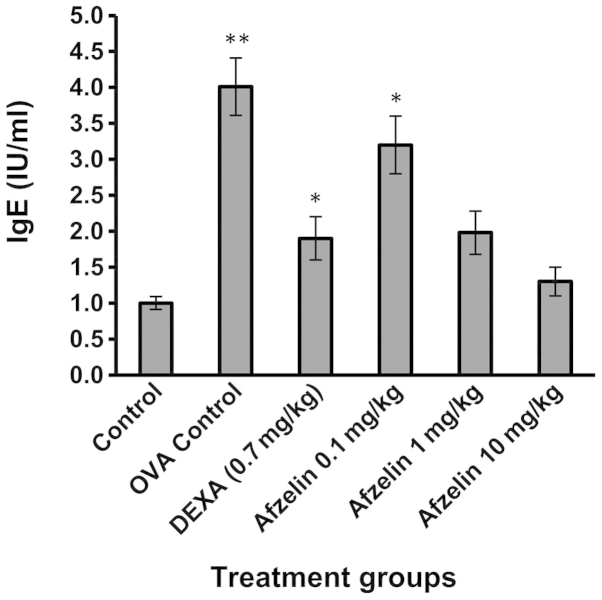
Effect of afzelin on OVA specific IgE release. Values are expressed as the mean ± standard error of the mean. ^*^P<0.05, vs. sham and ^**^P<0.01, vs. sham (Student’s t test). OVA, chicken egg albumin; IgE, immunoglobulin E.

OVA-specific IgE levels were elevated in the OVA group when compared with those in the control group (Fig. 6). Treatment with afzelin (1 and 10 mg/kg) demonstrated no significant change in OVA-specific IgE levels as compared with those in the control group.

### Afzelin alters the expression of T-bet and GATA-3 in the lungs

**Figure 7:**
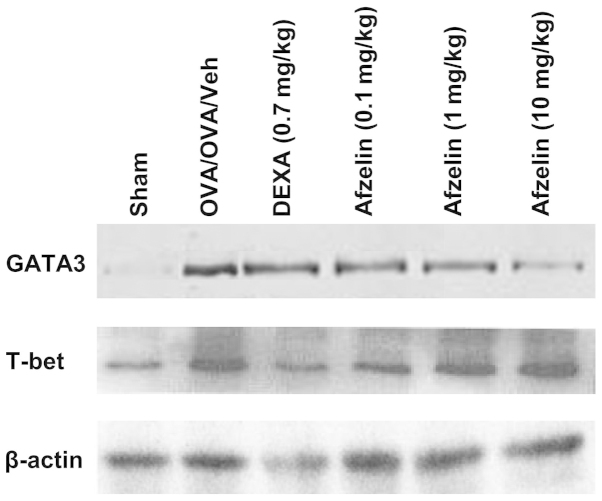
Effect of the test compounds on GATA3 and T-bet expression using western blot analysis. Dexa, dexamethasone; OVA, chicken egg albumin; GATA3, GATA-binding protein 3.

Expression levels of T-bet and GATA-3 in the lungs were altered in OVA control mice, Dexa-treated mice and afzelin-treated mice. However, no change was observed in the expression levels of any of these proteins in control animals. Treatment with afzelin increased expression of T-bet, while at the same time decreasing GATA-3 expression in a dose-dependent manner (Fig. 7).

## Discussion

In the present study, the effects of afzelin on allergen-induced airway inflammation and immune response in acute experimental asthma were assessed. It was found that administration of afzelin markedly reduced Th2 cytokine levels and OVA-specific IgE, and suppressed airway inflammatory cell infiltration induced by allergens, resulting in a decreased number of eosinophils and total inflammatory cells in BALF. Lung histology validated the effect of afzelin on airway inflammation. These findings suggested that afzelin is an anti-asthmatic agent and may be beneficial for the treatment of allergic asthma.

It is widely accepted that T cells have an important role in the injurious lung immune responses associated with asthma (13,14). CD4^+^ Th cells can be divided into Th1 and Th2 groups, functionally based on the various types of cytokine they produce. The different patterns of T-cell differentiation generate the different inflammatory effectors and those inflammatory effectors are correlated with the extent and type of damage observed in the airways (15–17). Under normal physiological conditions, the ratio of Th1 to Th2 cells is maintained at an appropriate level. Once the balance between Th1 and Th2 is disrupted, disease may occur (18). The two major Th-specific transcription factors T-bet and GATA-3, which regulate the expression of the cytokine genes, are characteristics of Th1 or Th2 and have crucial roles in Th-cell differentiation. It has been reported that a change in the T-bet/GATA-3 ratio reflects a change in the Th1/Th2 balance (18–20). Therefore, the T-bet/GATA-3 ratio may be used to evaluate the immune balance of Th1/Th2 responses in asthma (4). In addition, increased IL-4 production is correlated with excessive Th2-cell responses and increased IFN-γ levels are associated with excessive Th1 cell responses (21). In the present study, the ratio of T-bet to GATA-3 decreased in the asthma group compared with that in the control group and was partly reverted in the afzelin treatment groups. At an equal pace, IL-4 production was depressed and IFN-γ levels increased in the treatment groups. This change was more prominent in the 1 and 10 mg/kg afzelin-treated groups. Treatment with Dexa had similar effects to those of 1 mg/kg afzelin. The pathophysiology of AHR is complex as numerous factors contribute to its development. Allergen-induced airway inflammation is important among these factors, and Th2 cytokines, particularly IL-4, are critical in allergic inflammation and development of AHR (22).

In the present study, no significant difference was found in baseline airway resistance among the six groups. The airway resistance generated by administration of Mch at 30–270 *μ*g/kg was significantly increased in the OVA group and the afzelin (0.1 mg/kg)-treated group. However, the control group, Dexa- and afzelin (1 and 10 mg/kg)-treated groups revealed a sharp decrease in airway resistance. In conclusion, the present study indicated that afzelin is promising as a beneficial medication for the treatment of asthma through ameliorating allergic responses.

## Figures and Tables

**Table I:** Effect of afzelin on total cell count and differential cell count.

Treatment	Total count (×10^4^/ml)	Differential count (%)			
		Macro	Mono	Eosino	Neutro
SHAM	3.1±1.1	51.3±5.5	49.6±4.5	–	–
OVA/OVA/Veh	49.2±10.0	7.4±1.1	13.6±4.0	61.7±4.9	12.9±3.3
Dexa (0.7 mg/kg)	23.5±8.3	26.8±7.6	31.9±13.0	22.3±11.0	8.0±3.8
Afzelin (0.1 mg/kg)	35.6±11.2	17.9±6.9	38.2±2.3	52.2±4.4	9.4±2.3
Afzelin (1 mg/kg)	17.7±6.3	38.9±3.3	32.2±2.7	15.9±1.5	5.3±1.2
Afzelin (10 mg/kg)	15.5±10.2	47.0±13.0	42.5±9.3	11.5±3.2	3.3+1.1

Values are expressed as the mean ± standard error of the mean. Dexa, dexamethasone; OVA, chicken egg albumin; Veh, vehicle.
